# Crude extract of *Desmodium gangeticum* process anticancer activity via arresting cell cycle in G1 and modulating cell cycle-related protein expression in A549 human lung carcinoma cells

**DOI:** 10.37796/2211-8039.1362

**Published:** 2022-06-01

**Authors:** Yuh-Fung Chen, Yi-Hsien Lu, Huei-Yann Tsai

**Affiliations:** a Department of Pharmacology, China Medical University, Taichung, Taiwan; b Department of Pharmacy, China Medical University Hospital, Taichung, Taiwan; c Department of Food Nutrition and Health Biotechnology, Asia University, Taichung, Taiwan

**Keywords:** *Desmodium gangeticum* (L.) DC, Anti-lung cancer, A549 human lung cancer cells, Cell cycle arrest, G1, Apoptosis

## Abstract

**Background:**

*Desmodium gangeticum (*L.)DC., which belongs to the Leguminosae family, has been used in Taiwan and other subtropical countries as an external medicine to remove blood stasis, activate blood circulation, and reduce inflammation. It has been reported to have antioxidant effects and improve inflammatory responses in rats stimulated by pro-inflammatory agents and induced gastric ulcers in experimental animals over the past few decades. This plant has also been used to treat parasitic infections, but there are no reports regarding its effects on lung cancer. Therefore, this study attempted to investigate its water crude extract (in abbreviation DG) on lung cancer cells.

**Methods:**

A549 human lung cancer cells were tested for survival using MTT, trypan blue, and propidium iodide. The effects of various concentrations of the crude extract of *D. gangeticum* (DG) (0.125~1 mg/ml) on the cell cycle and apoptosis of A549 cells were analyzed by flow cytometry and Western blotting methods.

**Results:**

DG can inhibit the growth of A549 human lung cancer cells in a concentration- and time-dependent manner. DG arrested A549 cells in the G1 phase by increasing the proteins expression of p21, p27, cyclin D1, and cyclin E. Additionally, DG decreased the expression of cyclin A, B1, and Cdc 2 (CDK1) proteins.

**Conclusions:**

DG demonstrated the anti-lung cancer activity by arresting the cell cycle in G1 via increasing the p21, p27, cyclin D1, cyclin E, and decreasing Cdc2, cyclin A, and B1 proteins expression in A549 human lung cancer cells.

## 1. Introduction

In Taiwan, cancer has been the number one killer of the top ten causes of death for the past 39 years, and lung cancer ranks first in mortality rate [1]. According to the 2021 cancer statistics in the United States, lung cancer also has the highest mortality rate among all cancers [2]. In recent years, the main treatment strategies for lung cancer are surgery, chemotherapy, radiation therapy, or combined therapy, but the original problems still cannot be solved entirely. For the past years, the research and development of botanical drugs have increased globally. For example, paclitaxel (Paclitaxel® or Docetaxel®), commonly used to treat lung cancer, is an active ingredient extracted from natural plants [3]. Herbal medicines for anti-cancer are often found to have antioxidant, anti-inflammatory, anti-bacterial, or anti-viral effects. In the case of limited western medicine treatment for many diseases, adjuvant therapy such as herbal medicine often has a specific effect; in addition to current chemotherapy, Chinese herbal medicine may have a reference value for lung cancer [4]. Therefore, this study aimed to explore some herbal plants in Taiwan that have inhibitory effects on lung cancer cells.

*Desmodium gangeticu* (L.) DC. is a low-altitude shrub plant growing in the middle and southern Taiwan. Its water crude extract is referred to as DG. The chemical composition of this plant has been studied [5,6]. A series of studies [7,8] discussed its pharmacological activity in experimental animals in alcohol-, histamine-, and aspirin-induced gastric ulcers; DG played a role in cell protection and reducing gastric acid secretion in peptic ulcers [9]. DG improved learning and memory defects in scopolamine-induced memory loss [10].

Additionally, it regulated blood sugar levels via increasing insulin secretion, lowering cholesterol and triglycerides, and increasing HDL [11]. The anti-inflammatory and analgesic effects of DG also have been reported [12,13]. Follow-up research showed DG inhibited viability on various cancer cells, especially on A549 human lung adenocarcinoma cells, and the effect was concentration- and time-dependent. Therefore, this study attempted to explore further the pharmacological activity and mechanism of its anti-lung cancer effect.

Normal human cells have double sets of DNA, but some cancer cells may have incomplete or multiple sets of DNA due to abnormal differentiation [14]. By measuring the content of DNA in cells, it is possible to understand the distribution of the cell cycle and understand the effect of drugs on the damage caused by the drugs on the cancer cells when they are processed on the cells [15]. The cell cycle can be mainly divided into several stages: Gap 0 (G0) phase, Gap 1 (G1) phase, Synthesis (S) phase, Gap 2 (G2) phase, and Mitosis (M) stage. The cell cycle regulation has a rigorous control mechanism, in which the substances that promote the cell cycle are responsible for the cyclin-dependent kinases (CDK) group. CDK is usually not activated and requires the presence and combination of cyclin. After the two form a cyclin/CDK complex, the cell can be advanced from this state to the next state. Different cyclins are produced at specific times in the cell cycle and then broken down at specific times [16].

Cyclin D stimulates resting cells (G0), entering the G1 phase. Mammals have three types of cyclin D: cyclin D1, D2, and D3. When cyclin D binds to CDK4/6 [17,18], the activated cyclin D/CDK4/6 complex, in turn, activates retinoblastoma (RB) protein [19]. Cyclin E binds to CDK2 following activation of cyclin D/CDK; when the cyclin E/CDK2 complex is activated, it promotes the G1 phase to the S phase [20,21]. After the cell enters the S phase, cyclin E is decomposed. CDK2 is released and combined with cyclin A to form a cyclin A/CDK2 complex [16] to continue the cell cycle. Cyclin B binds to Cdc2 (CDK1) during cell division to control cell entry and termination of mitosis. Cyclin B is broken down when cell division is terminated and enters another cycle [16].

In recent years, studies have found that two protein families, KIP and INK4, can inhibit the activity of CDK, which is called CKI (CDK inhibitory protein), and KIP (kinase inhibitory protein) family includes: p21, p27, and p57. Inactivating it, if KIP is expressed in excess, the cells will stay in the G1 phase, and P53 can induce p21 to generate [22]. It has also been found that increased expression of p21 can cause cells to be quiescent in the S phase [23]. P27, on the other hand, inhibits the activity of cyclin/CDK in the G1 phase [24] and binds to cyclin A/CDK2 [25], resulting in cell cycle quiescence.

By inhibiting specific pathways, cells repair or inspect the condition of the cell itself and changes in the surrounding external environment. For example, if the cell cannot perform repair work, it will inhibit the activity of CDK and cyclin through CKI, stop the cell cycle, or go to apoptosis [26]. In order to avoid the occurrence of cell mutation or cancer caused by the transmission of wrong information of cells [27], this mechanism can be developed as a method for cancer treatment alone or as an adjuvant.

## 2. Materials and methods

### 2.1. Plant identification and crude extract from Desmodium gangeticum (L.) DC. (Abbreviated as DG)

*D. gangeticum* (L.) DC. was collected from middle Taiwan, and plant identification was performed by Dr. Yung-Chung Hsieh, Department of Pharmaceutical Sciences and Chinese Medicine Resources, China Medical University. Added 10 L of distilled water into dried leaves and stems of *D. gangeticum*, the extracts were collected (500 g), boiled, and steamed to the concentrate of 300 ml, and filtered the concentrate with a 0.22 mm filter membrane to get the final extract. The extract was evaporated to dryness and its weight was 27g (DG). The yield rate was 5.4%. DG was stored in a −80 °C freezer until use. Various concentrations of DG (0.125–1 mg/ml) were used to treat A549 cells.

### 2.2. Chemicals and reagents

RPMI-1640 cell culture medium, FBS (fetal bovine serum), Penicillin/streptomycin (P/S), and trypan blue were purchased from Gibco BRL, CA, USA. Dimethyl sulfoxide (DMSO), Propidium iodide (PI), Ribonuclease A (RNase A), Bovine serum albumin (BSA), Ammonium persulfate (APS), 3-(4,5-dimethylthiazol-2-yl)-2,5-diphenyl-tetrazolium bromide (MTT), Cellytic ™ cell lysis reagent, Triton X-100 were from Sigma–Aldrich Chemical company, St. Louis, USA. N–N–N’-N’-Tetramethylene-diamine (TEMED), Polyoxyethylenesorbitan monolaurate (Tween-20), trypran-EDTA, 40% acrylamide/BIS-ACRYLAMIDE (29:1 Ratio), 10X Sodium dodecylsulfate (SDS) buffer, Tris (tris(hydroxymethyl)-aminomethane), 10X SDS-PAGE running buffer were from Amersco company, USA. Apoptosis detection kit (PI/Annexin V-FITC) was from Becton Dickinson, USA. Western lightning chemiluminescene reagent plus (ECL) kit was from NEN Life Science, USA. Anti-cyclin A (ab7956), anticyclin B1 (ab2949), anti-cyclin D1 (ab7958), anticyclin E (ab7959), anti-CDk1/Cdc2 (ab6537), anti-Cdk2 (ab7954), anti-Cdk4 (ab7955), Anti-Cdk6 (ab41870), Anti-p27K1P1 (ab47590), Anti-p21 (ab47452), goat anti-mouse IgG (HRP) horseradish peroxide conjugated antibody were from Abcam company, Cambridge, UK. β-actin (C4): sc-477778, were from Santa Cruz biotechnology, CA, USA.

### 2.3. Cell culture

A549 human lung cancer cell line was purchased from the Bioresource Collection and Research Center (BCRC) in Hsinchu, Taiwan. A549 cells were maintained in RPMI-1640 medium containing 100 ml/L FBS and 100,000 U/L penicillin/100 mg/L streptomycin.

### 2.4. Cell viability

A549 cells were plated onto 96-well plates and incubated with various concentrations of DG (0.125–1 mg/ml) for 24–72 h. MTT was added to each well and incubated for another 1 h at 37 °C, then dissolved the blue formazan product in 200 μl DMSO for 15 min, and the plates were read using a spectrophotometric plate reader (Bio-Rad, Japan) at O.D.570 nm.

### 2.5. Cell cycle distribution and apoptosis determination

A549 cells were plated onto 12-well plates and incubated with various concentrations of DG (0.125–1 mg/ml) for 24–72 h. A549 cells were fixed in 75% ethanol at 4 °Cfor overnight and re-suspended in 1X PBS containing PI (40 mg/ml), RNase (0.1 mg/ml) and Triton X-100 (0.1%) for 30 min. Cell cycle distribution and apoptosis determination were analyzed by flow cytometry (FACS Calibur, Becton Dickinson, USA) as previously described [27]. After stimulation with DG (0.5 mg/ml, 1 mg/ml) for 24–72 h, the cells were stained with FITC conjugated Annexin-V/PI. The percentage of Annexin-V/PI stained cells was determined by flow cytometry. It was divided into a quadrant: (Q1) necrotic cells, (Q2) late apoptotic cells, (Q3) living cells, (Q4) early apoptotic cells.

### 2.6. Western blotting

A549 cells were plated onto 10-cm dishes and treated with various concentrations of DG (0.125–1 mg/ml) for 48 h. Total cell lysates were prepared as previously described [13]. Forty μg of total protein was applied to SDS-PAGE and transferred onto a polyvinylidene difluoride (PVDF) membrane. Then, the blots were incubated with the appropriate dilution of specific monoclonal antibodies for cyclin E, cyclin A, CDK2, Cdc 2, p21, p27, and Bcl-2. Protein expression was detected using enhanced chemiluminescence kits (ECL kits) [28].

### 2.7. IC50 calculation and statistical analysis

IC50 values of DG were calculated by linear approximation regression of the percentage survival versus the drug concentration. The experiments were performed at least in triplicate and all data were expressed as the mean ± standard error. Student’s *t*-test and one-way ANOVA followed by Dunnett’s test were used for single variable comparison and multiple variable comparisons, respectively. Significance was considered when *P < 0.05.

## 3. Results

### 3.1. Effects of DG on the morphology, viability, and apoptosis of A549 cells

**Fig. 1:**
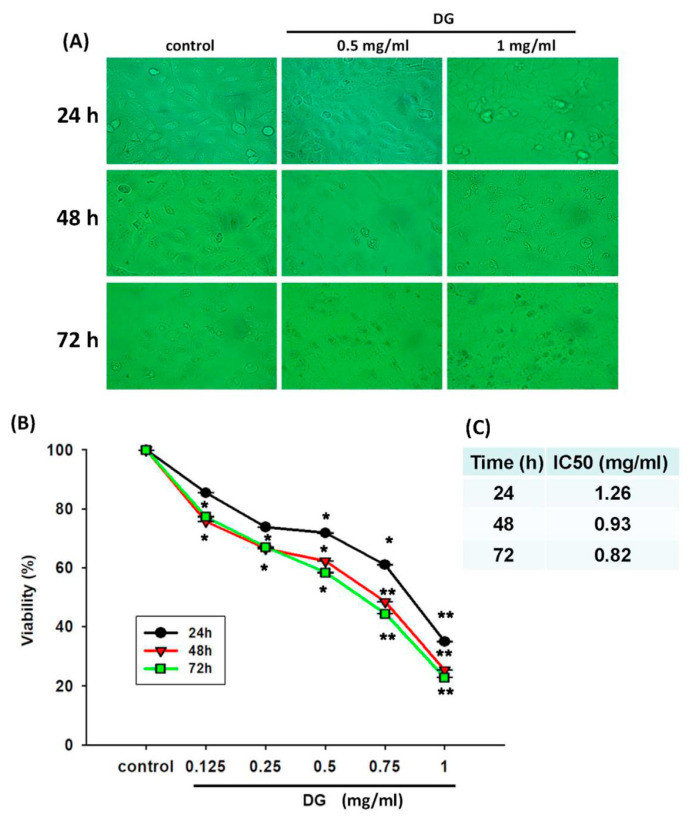
After DG treatment, the morphology and viability change of A549 human lung cancer cells. (A) The morphology changes of A549 cells after treatment with various DG concentrations (0.5 and 1 mg/ml) for 24, 48, and 72 h, respectively. (B) The viability of A549 was treated with various concentrations of DG (0.125~1 mg/ml) for different time courses (24, 48, and 72 h) by MTT viability assay. DG showed a concentration-dependent and time-dependent inhibition on the morphology and cell viability of A549 cells. (C) The IC 50 of DG at different time of treatment. Results represented as mean ± S.E. *P < 0.05, **P < 0.01 was considered statistically significant compared to the control group.

**Fig. 2:**
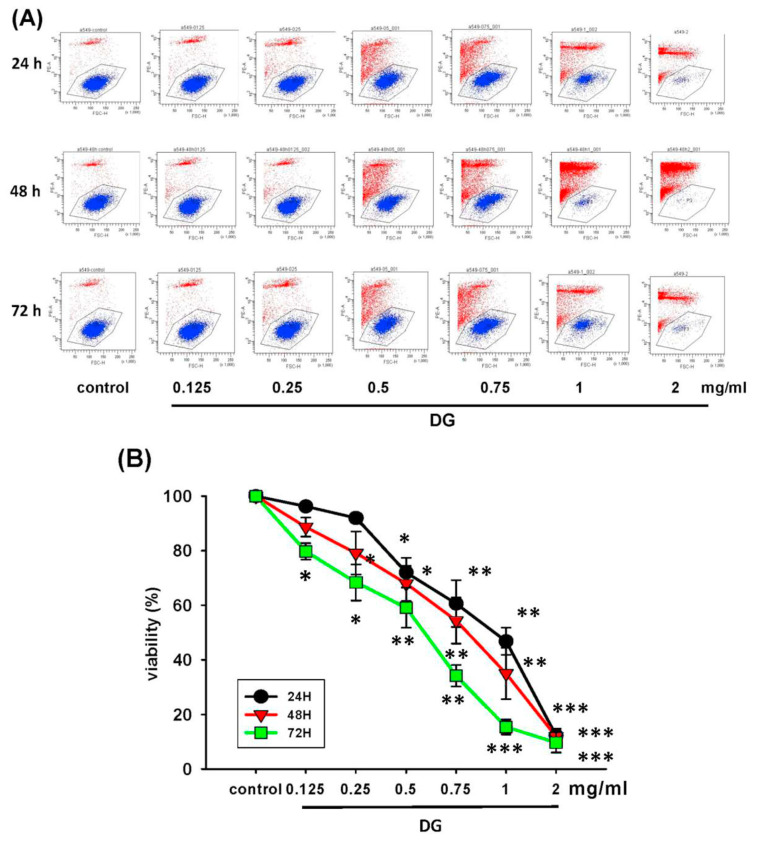
The cell viability of A549 cells treated with various concentrations of DG (0.125–2.0 mg/ml) for different time lengths. (A) The viability of A549 cells treated with various concentrations of DG for 24, 48, 72h, respectively, by flow cytometry. (B) The viability change and time course of A549 cells treated with various concentrations of DG for 24 to 72h. A549 Cell viability was detected by propidium iodide staining and expressed as % of control. DG inhibited the cell viability in a concentration-dependent manner by flow cytometry analysis. Data were expressed as mean ± S.E. *P < 0.05, **P < 0.01, and ***P < 0.001 was considered statistically significant compared to the control group.

**Fig. 3:**
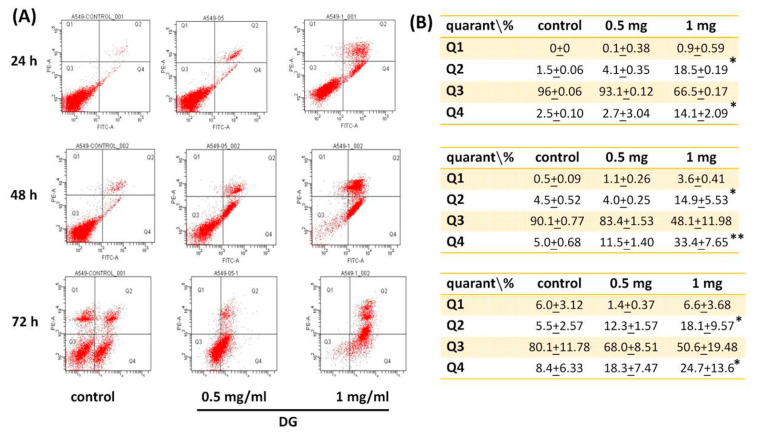
DG treatment-induced A549 lung cancer cells apoptosis. (A) A549 cells were treated with various concentrations of DG (0.5, 1 mg/ml) for different time courses (24h, 48h, and 72 h). After DG treatment, A549 cells were stained with FITC conjugated Annexin-V/PI. The percentage of Annexin-V/PI stained cells was determined by flow cytometry. It was divided into a quadrant: (Q1) necrotic cells, (Q2) late apoptosis cells, (Q3) living cells, (Q4) early apoptotic cells. (B) Represented the FITC conjugated Annexin-V/PI positive cells in each quadrant. DG showed a concentration- and time-dependent-induction of A549 cells apoptosis. Values are expressed as mean ± S.E. *P < 0.05, **P < 0.01 was considered statistically significant compared to the control group.

A549 cells were treated with various concentrations of DG (0.125, 0.25, 0.5, 0.75, and 1 mg/ml) for 24 h, 48h, and 72h, respectively, and the cell viability was detected by MTT tests and propidium iodide staining. DG showed a concentration-dependent and time-dependent inhibition on the morphology and cell viability of A549 cells, *P < 0.05 and **P < 0.01 as shown in Fig. 1. The IC50 of DG was 1.26 mg/ml, 0.93 mg/ml, and 0.82 mg/ml for 24, 48, and 72 h, respectively. The cell viability of DG-treated A549 cells detected by propidium iodide staining revealed that DG inhibited the cell viability in a concentration-dependent manner by flow cytometry analysis (as shown in Fig. 2). DG treatment-induced A549 cells apoptosis. After DG treatment, A549 cells were stained with FITC conjugated Annexin-V/PI, and the Annexin-V/PI stained cells were determined by flow cytometry. DG showed a concentration-dependent and time-dependent induction apoptosis of A549 human cells as shown in Fig. 3.

### 3.2. Effects of DG on the cell cycle of A549 cells

**Fig. 4:**
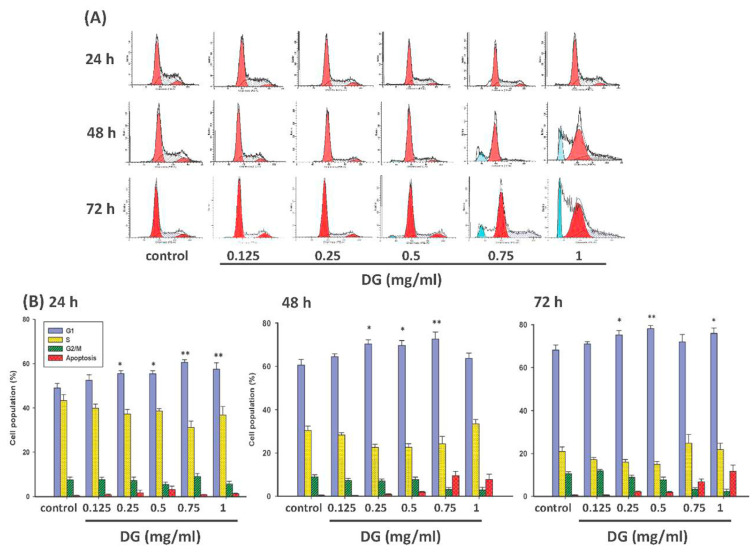
Flow cytometric analysis of cell cycle in A549 lung cancer cells after various concentrations of DG (0.125~1 mg/ml) treatment for different time courses (24~48 h). (A) The flow cytometry charts of DG-treated A549 cells. DG increased the G1 phase and reduced the S phase concentration and time-dependent. (B) Represented cell cycle distribution of control and DG-treated A549 cells as measured by propidium iodide staining of DNA at 24h, 48h, and 72 h, respectively. After treatment with various concentrations of DG (0.25~1 mg/ml) in A549 cells, DG increased the cell population in G1. When *P < 0.05 or **P < 0.01 is regarded as statistical significance in the proportion of cells in the G1 phase versus control cells.

After various concentrations of DG (0.125–1 mg/ml) treatment for different time courses (24–72 h), the cell cycle of A549 cells was analyzed by flow cytometry. The DNA content of the cell cycle is highest in the G1 phase, followed by the S phase, and the G2/M phase is the least. Compared to the control group, DG induced the G1 phase accumulation (*P < 0.05 for 0.25-l and 0.5 mg/ml and **P < 0.01 for 0.75 mg/ml) as shown in Fig. 4.

### 3.3. Effect of DG on the cell cycle-related proteins of A549 cells

**Fig. 5:**
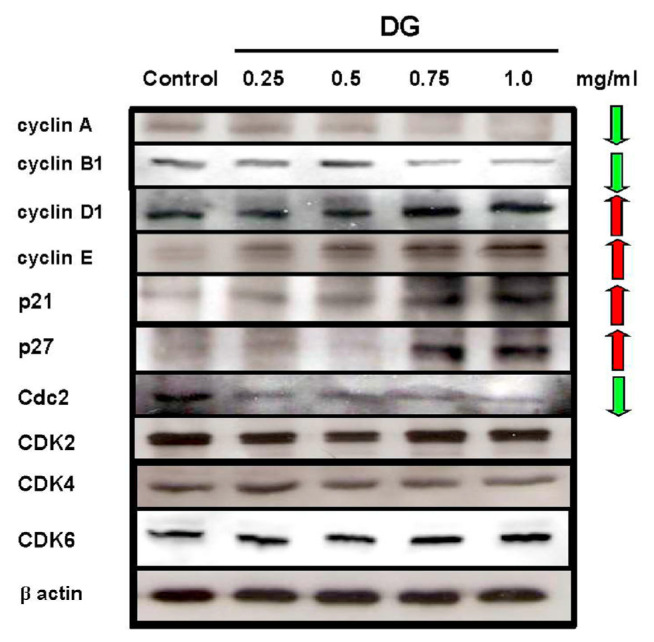
DG treatment for 48 h affected the protein expression of various cell cycle-related kinases in A549 lung cancer cells. Various concentration of DG (0.25~1 mg/ml) was used. The expression of cyclin A, cyclin B1, and Cdc2 were decreased after DG treatment; however, the expression of cyclin D1, cyclin E, p21, and p27 was increased after DG treatment.

After treatment with various concentrations of DG (0.25–1 mg/ml) in A549 cells, DG increased the cell population in G1. DG decreased the expression of cyclin A, cyclin B1, and Cdc2 (CDK1) proteins related to the S phase; and increased the expression of p21, p27, cyclin D1, and cyclin E proteins related to the G1 phase of the cell cycle in A549 cancer cells in a concentration-dependent manner as shown in Fig. 5.

## 4. Discussion

This study showed that DG had a concentration-dependent inhibitory effect on the survival rate of A549 human lung cancer cells. In cell morphology, after DG treatment, the cell morphology, cell membrane, and nucleus were ruptured.

In the early stage of cell apoptosis, phosphatidylserine in the cell membrane is externalized to the outside, one of the changes in early cell apoptosis. Annexin V is a calcium-dependent phospholipid-binding protein with a high affinity to phosphatidylserine. FITC is labeled on Annexin V, double-stained with PI, detected and analyzed by flow cytometry, distinguishing normal, necrotic, and apoptotic cells [29,30]. The results showed that DG also partially caused apoptosis in a concentration-dependent manner.

There is a very tight control mechanism in regulating the cell cycle. Different cyclins are produced at specific times in the cell cycle and then broken down at specific times [16]. Once a cell is damaged, some cell cycle-related proteins will activate and terminate the cell cycle and repair DNA to continue DNA replication and cell division. If repair is still impossible, cells will choose cell cycle quiescence, apoptosis [27]. The results of this experiment were analyzed by flow cytometry. It was found that cells in G0/G1 phase increased and decreased in the S phase after DG treatment, indicating that DG can induce the cell cycle of A549 human lung adenocarcinoma cells to arrested in the G1 phase.

The related regulatory protein cyclin regulates the cell cycle from one stage to another. The substances promoting the cell cycle are responsible for the regulatory protein kinase cyclin-dependent kinases (CDK) group. CDK is usually not activated. Instead, cyclin/CDK complexes are formed before the cells enter the next state. Different cyclins are produced and then decomposed at specific times in the cell cycle [16], so the operation of the cell cycle requires the interaction of its related proteins to complete. Different drugs will inhibit different cyclins and make cells quiescent in different periods to inhibit the growth of cancer cells. Cell cycle-specific drugs such as antimetabolites are most cytotoxic in the S phase, and bleomycin causes cell cycle quiescence in the G2 phase. Vinca alkaloids quiescent in metaphase (M), etoposide blocks the S/G2 phase of the cell cycle. Mechlorethamine and cisplatin often cause damage in the G1 and S phases of the cell cycle [31]. After DG treatment, A549 cells terminated in the G1 phase. The expression of cyclin A and Cyclin B1, cell cycle-related proteins, was inhibited, whereas the expression levels of cyclin D1 and cyclin E increased. The KIP and INK4 family of proteins can inhibit the activity of CDK, which is generally called CDK inhibitory protein (CKI). The kinase inhibitory protein (KIP) family includes p21, p27, and p53. P21 and p27 bind to the Cyclin/CDK complex, making it inactive. When KIP is overexpressed, cells will stay in the G1 phase. It has also been found that the expression of p21 can cause cells to be quiescent in the S phase [23]. The increase of p27 will inhibit the activity of cyclin/CDK in the G1 phase [24], and it will also bind to cyclin A/CDK2 [24], making it impossible to continue the operation of the cell cycle and inhibit cell growth.

This study revealed that the protein expression of p21 and p27 increased with the increase of DG concentration, inhibiting their growth and resulting in the inactivity of the cell cycle of A549 lung adenocarcinoma cells.

## 5. Conclusions

**Fig. 6:**
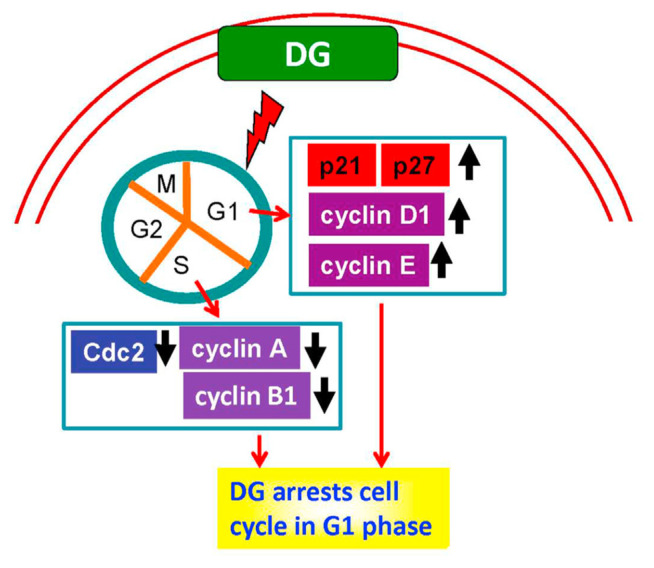
The anti-lung cancer mechanism of DG on A549 human lung cancer cells is proposed in this diagram. DG arrests cell cycle in the G1 phase via increasing protein expression of p21, p27, cyclin D1, and cyclin E; whereas DG decreases the expression of cyclin A, cyclinB1, and Cdc2, leading to apoptosis of A549 cells.

This experiment found that DG, a crude extract of *D. gangeticum*, could inhibit the growth of A549 human lung adenocarcinoma cells. DG decreased cyclin A, cyclin B1, and Cdc2 expression, whereas increased p21 and p27 expression in a concentration-dependent manner, thus, leading to arrest of the cell cycle in the G1phase as shown Fig. 6. Data implies that there will be opportunities to develop DG to an anticancer drug or adjuvant preparations for other anticancer drugs in the future.
